# Epigenetic regulation on left atrial function and disease recurrence after catheter ablation in atrial fibrillation

**DOI:** 10.1186/s13148-024-01794-9

**Published:** 2024-12-18

**Authors:** Mi-Ryung Han, Joo Hee Jeong, Yun Gi Kim, Hyun-Ho Yang, Chang-Ok Seo, Yeji Kim, Hyoung Seok Lee, Jaemin Shim, Young-Hoon Kim, Jong-Il Choi

**Affiliations:** 1 Division of Life Sciences, College of Life Sciences and Bioengineering, Incheon National University, Incheon, Republic of Korea; 2 Division of Cardiology, Korea University College of Medicine, Korea University Anam Hospital, 73 Goryeodae-ro, Seongbuk-gu, Seoul, 02841 Republic of Korea

**Keywords:** Atrial fibrillation, Epigenetics, Catheter ablation, Recurrence

## Abstract

**Background:**

Genetic variation and modifiable risk factors play a significant role in the pathogenesis of atrial fibrillation (AF). The influence of epigenetic modification on AF remains to be elucidated. We investigated the role of DNA methylation in the etiology of AF. Epigenetic evaluation was performed in 115 AF patients who underwent radiofrequency catheter ablation in a single institution. We measured methylation at approximately 850,000 bp cytosine-phosphate-guanine (CpG) sites in the 115 samples. The degree of methylation was compared across seven classification criteria: type of AF, late recurrence, impaired left atrium (LA) function, late gadolinium enhancement, LA diameter, LA volume, and flow velocity of the LA appendage.

**Results:**

The four most significantly methylated genes were *DEFB104B*, *C3*, *TANC1*, and *TMEM9B*. The *DEFB104B* gene (cg20223677 in the transcription start site), which encodes β-defensin 104B, was hypomethylated in three groups: AF patients with late recurrence, impaired LA function, and impaired LAA flow velocity. Enriched functional annotation of the differentially methylated datasets revealed that five out of the seven AF groups in this cohort were associated with genes involved in the cell movement of endothelial cell lines, sprouting angiogenesis by endothelial cell lines, or migration of endothelial cell lines.

**Conclusions:**

Epigenetic profiling revealed that epigenetic modification might affect important characteristics of AF. Our results suggest that the pathogenesis of AF might be affected by not only genetic variation or modifiable factors but also by epigenetic modulation.

## Introduction

Atrial fibrillation (AF) is associated with significant impairment in quality of life and various major adverse cardiovascular events. Due to the aging of the general population, the incidence and global medical burden of AF is rising rapidly [1]. Treatment of AF requires understanding of its pathophysiology. Clinical risk factors for AF include age, sex, lifestyle habits, and cardiovascular comorbidities [2, 3]. Genetic predisposition is another important risk factor for the development of AF. Previous studies identified several genetic variations that are potentially responsible for the occurrence of AF [4–6]. In addition, alteration in DNA methylation was suggested to play an important role in AF pathogenesis and relevant cardiovascular diseases [7, 8].

In a subset of patients, AF presents at a relatively young age with familial clustering [9]. It is assumed that the role of genetic factors is particularly pronounced in early-onset AF. Furthermore, the severity and characteristics of AF is highly heterogenous among affected patients. In some patients, atrial myopathy serves as an important substrate for AF maintenance. In others, ectopic beats from pulmonary veins are the main triggering source of AF [10, 11]. The underlying pathophysiology of AF still remains unclear and is multifactorial. Variations in DNA sequences and environmental risk factors are both likely to be involved in the pathogenesis of AF [12]. Epigenetic regulation in AF might complement the effects of genetic variations and environmental risk factors. However, little is known about the role of epigenetic regulation in AF development and progression. Therefore, we performed a genome-wide methylation analysis to examine the role of DNA methylation in the etiology of AF.

## Methods

### Study population and definition of variables

This study was based on a single-center cohort of AF patients that was treated with radiofrequency catheter ablation (RFCA) between 1998 and 2021 (Korea University Anam Hospital). Blood samples were obtained before performing RFCA, from 115 patients who gave informed consent for genetic testing. Patients were classified based on seven parameters that reflect disease severity in our prior study [13]: (i) Non-paroxysmal AF vs. paroxysmal AF, (ii) Late recurrence (positive vs. negative), (iii) Impaired left atrial function (positive vs. negative), (iv) Late gadolinium enhancement (≥ 20% vs. < 20%), (v) Left atrium (LA) diameter (≥ 45 mm vs. < 45 mm), (vi) LA volume (≥ 90 ml vs. < 90 ml), and (vii) Left atrial appendage (LAA) flow velocity (≤ 40 cm/sec vs. > 40 cm/sec).

Late recurrence was defined as any atrial tachyarrhythmia lasting more than 30 s that occurred after three months of RFCA. The extent of late gadolinium enhancement was obtained from contrast-enhanced cardiac magnetic resonance imaging during wash-out period. Left atrial function was assessed by transthoracic and transesophageal echocardiography before RFCA. Impaired LA function was defined as meeting any of the following criteria: (i) LA diameter ≥ 45 mm; (ii) LAA flow velocity ≤ 40 cm/sec; or (iii) Spontaneous echo contrast in the LAA. Spontaneous echo contrast is an echogenic swirling of blood flow during transesophageal echocardiography, which is a common indicative of blood stasis.

Informed written consent was obtained from all participants. The Institutional Review Board of Korea University Medicine Anam Hospital approved this study (IRB No. 2017AN0127). This study adhered the legal regulations of South Korea and the ethical guidelines of the 2013 Declaration of Helsinki throughout the study.

### Whole-methylome profiling

We used the Illumina Infinium Methylation EPIC Beadchip array (Illumina, San Diego, CA, USA) to measure methylation at approximately 850,000 bp cytosine-phosphate-guanine (CpG) sites in each of the 115 samples. Methylation status was represented as the beta (β) value, the ratio between methylated probe intensity and total probe intensity (sum of methylated and unmethylated probe intensities) that ranges from 0 to 1 and represents the proportion of methylation at each CpG site.

DNA samples were checked using a NanoDrop® ND-1000 UV–Vis Spectrophotometer (NanoDrop Technologies, Wilmington, DE, USA), and intact genomic DNA was diluted to 50 ng/μl using Quant-iT Picogreen quantitation (Invitrogen, Carlsbad, CA, USA). All prepared samples were bisulfite-converted with 500 ng of input gDNA according to the Zymo EZ DNA methylation kit protocols (Zymo Research, Irvine, CA, USA).

After the bisulfite-treated samples were amplified, the product was fragmented using a proprietary reagent (FMS), precipitated with 2-propanol (plus the precipitating reagent PM1), and resuspended in formamide-containing hybridization buffer (RA1). DNA samples were denatured at 95 °C for 20 min, then placed in a humidified container for a minimum of 16 h at 48 °C to allow the CpG loci to hybridize onto the EPIC Beadchip. The arrays were washed and scanned using the Illumina iScan platform.

### Genome-wide DNA methylation analysis

For each probe, signal intensities were extracted using Illumina’s iScan Control software. Data analysis was performed in R software using the Chip Analysis Methylation Pipeline package from Bioconductor [14]. The “minfi” R package provides quality control and normalization options for the Illumina Intensity Data files that contained the raw intensity signals from the red and green color channels [15]. Samples were excluded if they failed to cluster with the others based on all probes using hierarchical clustering or if they showed lower median intensities in methylated or unmethylated signals. Probes with a signal detection p-value > 0.01 in at least one sample, probes with < 3 beads in at least 5% of the sample, single-nucleotide polymorphism–related probes, non-CpG probes, cross-reactive probes, sex chromosome probes, and multi-hit probes were also removed. Beta‐mixture quantile normalization was used to adjust the processed data for bias introduced by the Infinium Type II probes. A single value decomposition analysis was conducted to detect technical batches and covariates, and then batch effects were corrected using combat algorithms according to the standard protocol before the differential methylation analysis. Combat algorithms use the parametric or non-parametric empirical Bayes method to adjust for potential batch effects [16]. β values were generated by dividing the methylated probe signal by the sum of the methylated and unmethylated signals at each CpG site. The value ranges from 0 (unmethylated) to 1 (fully methylated).

Differentially methylated positions (DMPs) were identified separately for the seven groups using linear regression methods from limma with age and sex as covariates [17]. We corrected for multiple testing using the false discovery rate according to the Benjamini–Hochberg procedure [18].

### Pathway and network analyses

Ingenuity Pathway Analysis (Ingenuity Systems, Redwood City, CA, USA) was used to perform the enriched function annotation and pathway analyses. Analyses were conducted on genes annotated to DMPs with a *p*-value < 0.05 and delta β > 0.05 or delta β < − 0.05 for the seven groups (an arbitrary cutoff for suggestive association). The delta β value is the difference in average β values between two sample groups. We did not use the more stringent criterion of a |delta β| of 0.15 or higher that we used in the DMP analyses because that included too few DMPs in the enrichment analyses. Networks were ranked using the score computed for each network according to the fit of the set of supplied focus genes in the Ingenuity Pathway Analysis. For each group, top pathways were identified with a canonical pathway (generalized pathway that represents the common properties of a particular signaling module or pathway) based on two parameters: (1) The ratio between the number of genes annotated to DMPs that map to the pathway and the total number of molecules that map to the canonical pathway, and (2) The *p*-value calculated using Fisher’s exact test to generate the probability that the association between genes annotated to DMPs and the canonical pathway was explained by chance alone.

### Validation of methylation status

We used a bisulfite pyrosequencing analysis to confirm the results obtained from the Illumina Infinium Methylation EPIC Beadchip array. Bisulfite-converted DNA was PCR amplified using primers designed in Pyrosequencing Assay Design Software v2.0 (QIAGEN). The methylation level was estimated using the M-value, which is the log2 ratio between the intensities of methylated probes and those of unmethylated probes. The *p*-value was calculated with and without the Benjamini–Hochberg false discovery rate method to correct for multiple comparisons.

## Results

### Patient characteristics

The baseline demographics of the patients are summarized in Table 1. In brief, AF was non-paroxysmal in 44 patients (38.3%). The mean age was 53.5 ± 16.5 years, and 95 patients (82.6%) were male. The mean CHA_2_DS_2_-VASc score were 1.5 ± 1.4. The mean LA diameter, left ventricular ejection fraction, and LAA flow velocity were 40.4 ± 6.0 mm, 55.1 ± 6.1%, and 52.7 ± 19.4 cm/sec, respectively. Differences among the seven classification criteria are also summarized in Table 1.

**Table 1:** Clinical characteristics of AF patients

	Whole cohort (n = 115)	AF type			Late recurrence			Impaired left atrial function		
		Non-paroxysmal (n = 44)	Paroxysmal (n = 71)	*p*	( +) (n = 26)	(-) (n = 89)	*p*	( +) (n = 44)	(-) (n = 71)	*p*
*Clinical findings*										
Non-paroxysmal AF	44	44	0		14	30	0.063	31	13	< 0.001
Age (year)	53.5 ± 16.5	56.9 ± 15.8	51.5 ± 16.7	0.087	45.0 ± 16.4	56.0 ± 15.8	0.003	59.2 ± 15.5	50.0 ± 16.2	0.03
Sex (male)	95	38	57	0.403	21	74	0.778	34	61	0.235
Body weight (kg)	73.1 ± 10.8	75.2 ± 12.6	71.7 ± 9.4	0.092	77.4 ± 13.3	71.8 ± 9.7	0.052	73.4 ± 11.5	72.9 ± 10.5	0.792
Height (cm)	169.3 ± 9.2	169.0 ± 9.1	169.5 ± 9.3	0.767	170.9 ± 10.3	168.8 ± 8.8	0.313	166.6 ± 9.5	171.0 ± 8.7	0.013
Body mass index (kg/m^2^)	25.5 ± 3.4	26.3 ± 4.0	25.0 ± 2.9	0.041	26.6 ± 4.5	25.2 ± 3.0	0.159	26.5 ± 3.8	24.9 ± 3.1	0.019
Heart failure	11	6	5	0.243	2	9	0.712	8	3	0.021
Hypertension	55	30	25	0.001	11	44	0.522	28	27	0.008
Diabetes mellitus	11	6	5	0.243	2	9	0.712	8	3	0.021
History of ischemic stroke, TIA, systemic embolism	11	5	6	0.606	0	11	0.059	5	6	0.606
Vascular disease	5	3	2	0.369	5	0	0.586	3	2	0.369
CHA_2_DS_2_-VASc	1.5 ± 1.4	1.9 ± 1.4	1.2 ± 1.3	0.005	1.0 ± 1.0	1.6 ± 1.5	0.038	2.1 ± 1.6	1.1 ± 1.2	< 0.001
*Echocardiographic findings*										
Left atrial diameter (mm)	40.4 ± 6.0	43.1 ± 5.0	38.8 ± 6.0	< 0.001	41.3 ± 7.2	40.2 ± 5.6	0.379	44.9 ± 5.3	37.6 ± 4.6	< 0.001
Left ventricular ejection fraction (%)	55.1 ± 6.1	54.1 ± 6.2	55.8 ± 5.9	0.133	54.5 ± 6.8	55.3 ± 5.9	0.574	54.3 ± 6.9	55.6 ± 5.5	0.275
E/e’	8.6 ± 3.5	9.4 ± 3.8	8.2 ± 3.3	0.068	7.9 ± 4.1	8.9 ± 3.3	0.203	9.9 ± 3.9	7.8 ± 3.0	0.004
Left atrial appendage flow velocity (cm/sec)	52.7 ± 19.4	40.4 ± 17.0	60.1 ± 16.8	< 0.001	46.7 ± 21.1	54.4 ± 18.6	0.075	35.2 ± 13.3	63.2 ± 14.2	< 0.001
Spontaneous echo contrast	14	11	3	0.002	9	5	0.211	14	0	< 0.001
Dense spontaneous echo contrast	2	2	0	0.144	0	2	> 0.999	2	0	0.144

	LGE*			LA diameter			LA volume*			LAA flow velocity		
	≥ 20% (n = 30)	< 20% (n = 77)	*p*	≥ 45 mm (n = 22)	< 45 mm (n = 93)	*p*	≥ 90 ml (n = 35)	< 90 ml (n = 74)	*p*	≥ 40 cm/sec (n = 29)	< 40 cm/sec (n = 86)	*p*
*Clinical findings*												
Non-paroxysmal AF	13	26	0.356	13	31	0.025	17	23	0.077	23	21	< 0.001
Age (year)	56.6 ± 18.9	52.7 ± 15.5	0.318	60.1 ± 16.1	52.0 ± 16.3	0.036	60.8 ± 15.2	51.1 ± 16.1	0.004	57.7 ± 16.5	52.1 ± 16.4	0.116
Sex (male)	25	62	0.737	16	79	0.174	6	14	0.823	22	73	0.268
Body weight (kg)	74.4 ± 8.5	7.7 ± 10.7	0.213	73.2 ± 11.6	73.0 ± 10.7	0.931	73.2 ± 12.0	72.7 ± 10.2	0.803	73.1 ± 12.8	73.0 ± 10.2	0.967
Height (cm)	170.6 ± 8.6	168.5 ± 9.3	0.27	165.4 ± 9.4	170.2 ± 8.9	0.024	167.9 ± 10.1	170.0 ± 8.6	0.357	167.1 ± 10.2	170.1 ± 8.8	0.128
Body mass index (kg/m^2^)	25.6 ± 2.7	25.3 ± 3.7	0.671	26.7 ± 2.7	25.2 ± 3.5	0.068	26.0 ± 4.2	25.2 ± 3.1	0.276	26.2 ± 4.3	25.3 ± 3.1	0.198
Heart failure	5	3	0.038	4	7	0.217	6	4	0.072	5	6	0.104
Hypertension	17	33	0.198	14	41	0.099	22	30	0.029	18	37	0.076
Diabetes mellitus	1	8	0.44	3	8	0.438	5	6	0.317	6	5	0.019
History of ischemic stroke, TIA, systemic embolism	2	9	0.724	1	10	0.688	6	5	0.093	4	7	0.371
Vascular disease	2	2	0.313	2	3	0.243	3	2	0.325	2	3	0.436
CHA_2_DS_2_-VASc	1.8 ± 1.4	1.3 ± 1.4	0.092	2.1 ± 1.7	1.3 ± 1.3	0.033	2.2 ± 1.4	1.2 ± 1.3	< 0.001	2.1 ± 1.4	1.2 ± 1.4	0.003
*Echocardiographic findings*												
Left atrial diameter (mm)	39.9 ± 6.6	40.2 ± 5.8	0.853	49.0 ± 3.8	38.4 ± 4.4	< 0.001	44.4 ± 6.2	38.5 ± 5.2	< 0.001	43.6 ± 5.7	39.3 ± 5.8	0.001
Left ventricular ejection fraction (%)	54.3 ± 7.6	55.9 ± 4.9	0.315	55.1 ± 6.2	55.1 ± 6.1	0.98	54.0 ± 8.2	55.9 ± 4.5	0.196	53.7 ± 7.8	55.6 ± 5.3	0.22
E/e’	8.7 ± 3.6	8.7	0.977	10.2 ± 4.0	8.3 ± 3.3	0.024	10.1 ± 4.3	8.1 ± 3.0	0.016	10.2 ± 4.2	8.1 ± 3.1	0.021
Left atrial appendage flow velocity (cm/sec)	56.3 ± 19.9	52.4 ± 19.5	0.358	41.4 ± 15.0	55.2 ± 19.4	0.003	43.8 ± 18.9	56.9 ± 18.5	0.001	27.9 ± 8.3	61.1 ± 14.1	< 0.001
Spontaneous echo contrast	3	8	1.000	16	6	0.016	3	5	0.006	12	2	< 0.001
Dense spontaneous echo contrast	0	1	> 0.999	2	0	0.035	2	0	0.101	1	1	0.442

*LGE or LA volume were assessed in patients with available data (107 and 109 patients, respectively)

*AF* Atrial fibrillation; *LA* Left atrial; *LAA* Left atrial appendage; *LGE* Late gadolinium enhancement; *TIA* Transient ischemic attack

### Differential methylation analysis

We analyzed 115 AF patients in seven different groups. After quality control procedures, 750,083 CpG sites remained and were used to identify DMPs. At a p-value < 0.05, the number of DMPs in each group was: (i) 43,398 hypermethylated CpG sites and 99,946 hypomethylated CpG sites in the non-paroxysmal AF vs. paroxysmal AF; (ii) 105,796 hypermethylated CpG sites and 45,522 hypomethylated CpG sites in the late recurrence ( +) vs. late recurrence (-); (iii) 28,343 hypermethylated CpG sites and 38,488 hypomethylated CpG sites in the impaired LA function ( +) vs. impaired LA function (-); (iv) 40,523 hypermethylated CpG sites and 59,445 hypomethylated CpG sites in the late gadolinium enhancement (≥ 20%) vs. late gadolinium enhancement (< 20%); (v) 35,537 hypermethylated CpG sites and 27,912 hypomethylated CpG sites in the LA diameter (≥ 45 mm) vs. LA diameter (< 45 mm); (vi) 35,235 hypermethylated CpG sites and 86,929 hypomethylated CpG sites in the LA volume (≥ 90 ml) vs. LA volume (< 90 ml); and (vii) 27,712 hypermethylated CpG sites and 62,592 hypomethylated CpG sites in the LAA flow velocity (≤ 40 cm/sec) vs. LAA flow velocity (> 40 cm/sec).

**Fig. 1:**
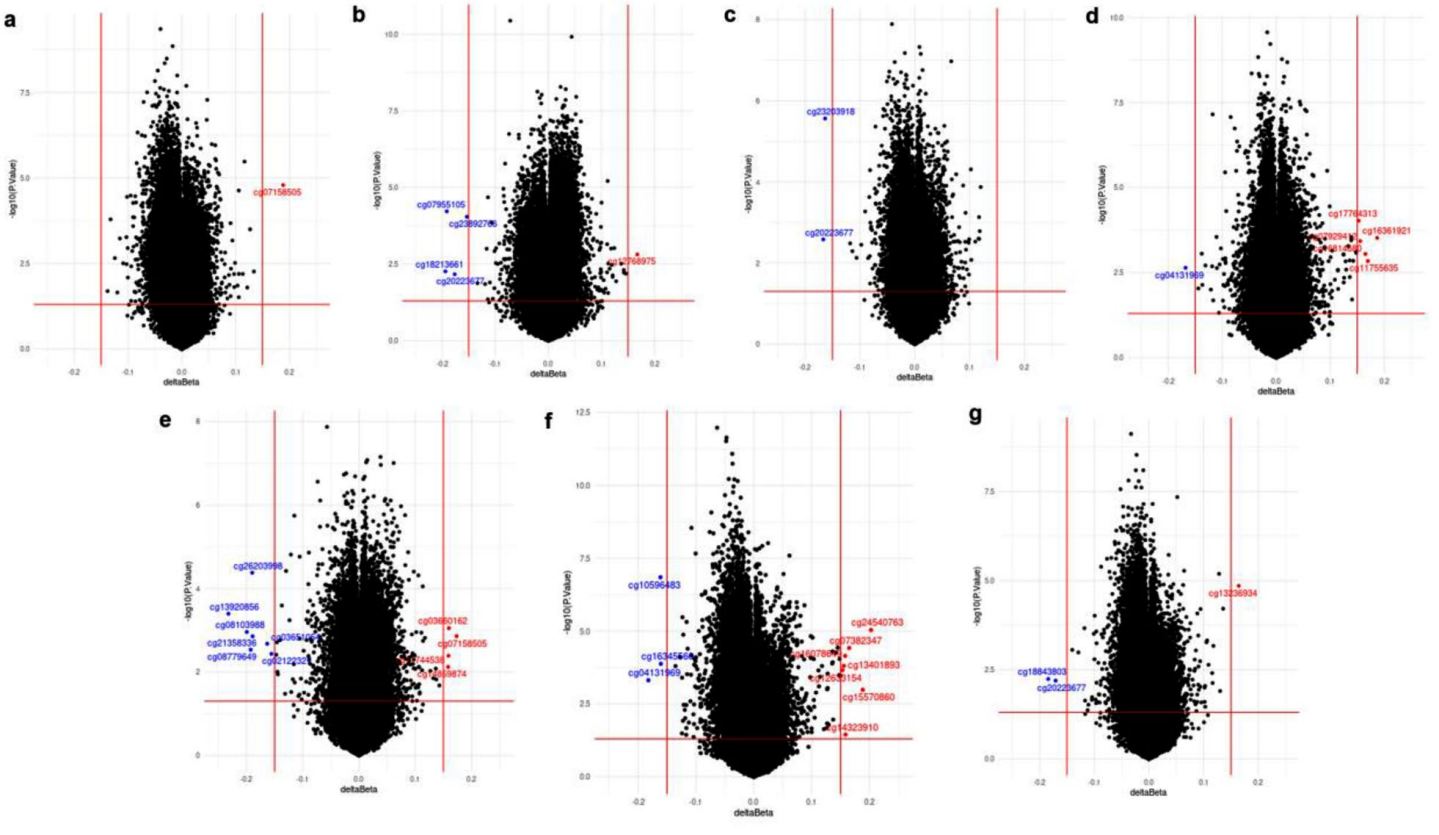
Volcano plots of differentially methylated CpG sites in the seven groups. Horizontal axis: the difference in average β values between the two conditions in each group (delta β); vertical axis: − log10 of the *p*-value. Vertical lines highlight delta β of − 0.15 and 0.15, and the horizontal line represents a *p*-value of 0.05. The red dots represent hypermethylated CpG sites, and the blue dots represent hypomethylated CpG sites. **a**. non-paroxysmal AF vs. paroxysmal AF; **b**. late recurrence ( +) vs. late recurrence (-); **c**. impaired LA function ( +) vs. impaired LA function (-); **d**. late gadolinium enhancement (≥ 20%) vs. late gadolinium enhancement (< 20%); **e**. LA diameter (≥ 45 mm) vs. LA diameter (< 45 mm); **f**. LA volume (≥ 90 ml) vs. LA volume (< 90 ml); **g**. LAA flow velocity (≤ 40 cm/sec) vs. LAA flow velocity (> 40 cm/sec). The data were based on blood samples from 115 patients. LA: left atrium; LAA: left atrial appendage

Using the criteria of p-value < 0.05 and |delta β|≥ 0.15, we identified 38 DMPs from the seven groups with an absolute change in average methylation (delta β) from 0.15 to 0.23 (Fig. 1). Some of the enriched DMPs were located in the gene body and intergenic regions, and the influence of DNA methylation in those regions is generally unknown. Regarding corresponding gene regions, seven (18%) DMPs were located in proximal promoter regions, including the TSS1500 (1500 bp upstream of the transcription starting site) and 5’UTR (5′ untranslated region) (Table 2). Among them, the four most significantly methylated genes in our cohort were DEFB104B, C3, TANC1, and TMEM9B. Interestingly, the DEFB104B gene (cg20223677 in the transcription start site) was hypomethylated in three groups: those with late recurrence, impaired LA function, and impaired LAA flow velocity. We found no significant difference in methylation at cg20223677 when comparing AF patients by age (older than 50 years vs. younger than 50 years) or sex (males vs. females) (data not shown).

**Table 2:** Differentially methylated CpG sites in AF patients

Group	CpG site	*p*-value	Adj. *p*-value*	Delta β*	Chromosome	Position	Gene	Genomic feature
Non-paroxysmal AF (*n* = 44) vs. Paroxysmal AF (*n* = 71)	cg07158505	1.61E−05	1.50E−02	0.19	18	51,612,914		IGR
Late recurrence ( +) (*n* = 26) vs. Late recurrence (-) (*n* = 89)	cg07955105	6.04E−05	1.77E−02	− 0.19	10	27,639,156		IGR
	cg23892766	9.08E−05	2.02E−02	− 0.15	10	27,639,136		IGR
	cg12768975	1.55E−03	5.67E−02	0.17	19	6,721,965	*C3*	TSS1500
	cg18213661	5.49E−03	9.33E−02	− 0.19	11	93,681,423		IGR
	cg20223677	6.80E−03	1.02E−01	− 0.18	8	7,332,846	*DEFB104B*	TSS1500
Impaired left atrial function ( +) (n = 44) vs Impaired left atrial function (-) (n = 71)	cg23203918	2.75E−06	2.99E−02	− 0.16	8	128,235,836		IGR
	cg20223677	2.62E−03	2.44E−01	− 0.17	8	7,332,846	*DEFB104B*	TSS1500
Late gadolinium enhancement (≥ 20%) (n = 30) vs Late gadolinium enhancement (< 20%) (n = 77)	cg17764313	9.44E−05	3.11E−02	0.15	3	127,335,263	*MCM2*	Body
	cg16361921	3.03E−04	5.17E−02	0.19	5	106,599,492		IGR
	cg07929412	3.77E−04	5.70E−02	0.16	2	130,694,084	*LOC101927924*	Body
	cg16814680	9.01E−04	8.09E−02	0.16	8	91,681,699		IGR
	cg11755635	1.45E−03	9.83E−02	0.17	12	8,762,304	*AICDA*	Body
	cg04131969	2.30E−03	1.18E−01	− 0.17	2	33,951,647	*MYADML*	Body
Left atrial diameter (≥ 45 mm) (n = 22) vs Left atrial diameter (< 45 mm) (n = 93)	cg26203998	4.15E−05	8.61E−02	− 0.19	6	183,775	*LOC285766*	Body
	cg13920856	4.00E−04	1.67E−01	− 0.23	2	159,867,184	*TANC1*	5’UTR
	cg03660162	8.86E−04	2.10E−01	0.16	16	78,913,085	*WWOX*	Body
	cg08103988	1.10E−03	2.24E−01	− 0.2	17	6,558,365		IGR
	cg07158505	1.38E−03	2.40E−01	0.17	18	51,612,914		IGR
	cg21358336	1.39E−03	2.40E−01	− 0.19	17	6,558,440		IGR
	cg03651054	2.10E−03	2.66E−01	− 0.16	13	50,194,643		IGR
	cg08779649	2.92E−03	2.88E−01	-0.19	13	50,194,554		IGR
	cg02122327	3.66E−03	3.06E−01	− 0.16	13	50,194,322		IGR
	cg11744538	4.08E−03	3.14E−01	0.16	17	42,646,995		IGR
	cg14859874	7.56E−03	3.71E−01	0.16	1	154,238,265	*UBAP2L*	Body
Left atrial volume (≥ 90 ml) (n = 35) vs Left atrial volume (< 90 ml) (n = 74)	cg10596483	1.43E−07	5.80E−04	− 0.16	8	143,751,796	*JRK*	TSS1500
	cg24540763	9.24E−06	4.48E−03	0.2	12	122,377,170	*WDR66*	Body
	cg07382347	3.80E−05	9.08E−03	0.16	6	30,039,408	*RNF39*	Body
	cg16078649	7.09E−05	1.24E−02	0.16	6	30,039,466	*RNF39*	Body
	cg16345566	1.33E−04	1.69E−02	− 0.16	6	32,633,102	*HLA-DQB1*	Body
	cg13401893	1.57E−04	1.83E−02	0.16	6	30,039,432	*RNF39*	Body
	cg12633154	2.19E−04	2.16E−02	0.15	6	30,039,435	*RNF39*	Body
	cg04131969	4.88E−04	3.21E−02	− 0.18	2	33,951,647	*MYADML*	Body
	cg15570860	1.04E−03	4.68E−02	0.19	11	8,986,840	*TMEM9B*	TSS1500
	cg14323910	3.58E−02	2.64E−01	0.16	6	32,628,305	*HLA-DQB1*	Body
Left atrial flow velocity (≤ 40 cm/sec) (n = 29) vs Left atrial flow velocity (> 40 cm/sec) (n = 86)	cg13236934	1.40E−05	2.33E−02	0.16	17	19,409,959		IGR
	cg18843803	5.74E−03	1.98E−01	− 0.18	19	31,799,406	*TSHZ3*	Body
	cg20223677	6.38E−03	2.06E−01	− 0.17	8	7,332,846	*DEFB104B*	TSS1500

*adj. *p*-value refers to Benjamini-Hochberg-adjusted *p*-value, and delta β refers to change in average methylation

*AF*: Atrial fibrillation; *IGR*: Intergenic region; Body: Gene body; CpG: Cytosine-phosphate-guanine; *TSS*: Transcription start site (1500-up to 1500 bp upstream from TSS); 5′ UTR: 5′ untranslated region

To confirm our observations, we chose to validate four CpG sites in proximal promoter regions (cg20223677 in the late recurrence, impaired LA function, and impaired LAA flow velocity groups, cg12768975 in the late recurrence group, cg13920856 in the LA diameter group, and cg15570860 in the LA volume group). Among them, cg20223677 was the most frequently hypomethylated probe (three of the seven groups). In the DEFB104B gene regions, differential methylation at cg20223677 was successfully replicated when comparing AF patients with and without impaired LA function (*p*-value = 0.026, log2 fold change = − 0.32). Although of similar magnitude and in the same direction, differential methylation at cg20223677 did not reach significance when comparing AF patients with and without late recurrence and AF patients who had high (≤ 40 cm/sec) or low (> 40 cm/sec) LAA flow velocity (*p*-value = 0.16, log2 fold change = − 0.22 and *p*-value = 0.45, log2 fold change = − 0.13, respectively). Due to a few structural issues and insertion alleles, we could not validate the other three CpG sites (cg12768975, cg13920856, and cg15570860).

### Functional enrichment analyses

In silico functional analyses were conducted to identify the potential biological functions associated with the genes that harbored DMPs, and found that biological pathways and networks were enriched in our association results.

The functions associated with AF patients in the seven groups were assigned based on genes annotated to DMPs (Table 3). Five of the seven groups in this cohort were associated with genes involved in the cell movement of endothelial cell lines, sprouting angiogenesis by endothelial cell lines, or migration of endothelial cell lines. Those groups were AF patients with non-paroxysmal or paroxysmal AF, with or without late recurrence, with or without impaired LA function, increased (≥ 90 ml) or decreased (< 90 ml) LA volume, and low (≤ 40 cm/sec) or high (> 40 cm/sec) LAA flow velocity. Familial cardiovascular disease and familial hypertrophic cardiomyopathy-6 were among the top hits in AF patients with increased (≥ 45 mm) or decreased (< 45 mm) LA diameter and low (≤ 40 cm/sec) or high (> 40 cm/sec) LAA flow velocity, respectively.

**Table 3:** Enriched functional annotations of the differentially methylated datasets

Group	Functional annotation	*p*-value	Genes	Number of genes
Non-paroxysmal AF (*n* = 44) vs. Paroxysmal AF (*n* = 71)	Sprouting angiogenesis	1.20E−03	*E2F7,ECT2,NRP1*	3
	Ataxia-telangiectasia	7.30E−03	*ESR1,PPARG*	2
	Cell movement of endothelial cell lines	9.90E−03	*CD44,CHI3L1,DLC1,ESR1,PPARG*	5
Late recurrence ( +) (*n* = 26) vs Late recurrence (-) (*n* = 89)	Heart block	9.40E−03	*C3,TRPM4*	2
	Progressive familial heart block type IB	9.90E−03	*TRPM4*	1
	Sprouting angiogenesis by endothelial cell lines	9.90E−03	*CDH13*	1
Impaired left atrial function ( +) (n = 44) vs Impaired left atrial function (-) (n = 71)	Primary ciliary dyskinesia type 28 with *situs inversus*	3.80E−03	*SPAG1*	1
	Augmented response of HDL cholesterol to hormone replacement	3.80E−03	*ESR1*	1
	Migration of endothelial cell lines	4.40E−03	*CHI3L1,ESR1,PLPP3*	3
	Hypertension	5.30E−03	*CYP2E1,ESR1,GALNT18,HAGH,MYO3B,PDE4C,RHOJ,RNF220,TMEM140*	9
	Binding of endothelial cells	5.60E−03	*CCR2,MUC4,NRP1,PLPP3*	4
Late gadolinium enhancement (≥ 20%) (n = 30) vs Late gadolinium enhancement (< 20%) (n = 77)	Dilated cardiomyopathy type 1JJ	6.10E−03	*LAMA4*	1
Left atrial diameter (≥ 45 mm) (n = 22) vs Left atrial diameter (< 45 mm) (n = 93)	Familial heart disease	5.20E−03	*CACNA1D,CACNA1E,CHRM2,DNAH5,HRH1,NOTCH1,PDLIM3,SGCD*	8
	Familial cardiovascular disease	6.70E−03	*C3,CACNA1D,CACNA1E,CHRM2,DNAH5,HRH1,NOTCH1,PDLIM3,PRKG1,SGCD,WNK4*	11
	Familial thoracic aortic aneurysm type 8	8.80E−03	*PRKG1*	1
	Dilated cardiomyopathy type 1L	8.80E−03	*SGCD*	1
	Aortic valve disease type 1	8.80E−03	*NOTCH1*	1
	Pseudohypoaldosteronism type IIB	8.80E−03	*WNK4*	1
	Sinoatrial node dysfunction and deafness	8.80E−03	*CACNA1D*	1
Left atrial volume (≥ 90 ml) (n = 35) vs Left atrial volume (< 90 ml) (n = 74)	Sprouting angiogenesis	1.00E−03	*CDH13,LOX,NRP1*	3
	Sprouting angiogenesis by endothelial cells	2.50E−03	*CDH13,LOX*	2
	Familial aortic disorder	7.30E−03	*GATA4,LOX,PRKG1*	3
Left atrial flow velocity (≤ 40 cm/sec) (n = 29) vs Left atrial flow velocity (> 40 cm/sec) (n = 86)	Sprouting angiogenesis	3.00E−04	*CDH13,LOXL2,NRP1*	3
	Hypertension	5.00E−04	*ADCY4,BOK,CDH13,CYB5R3,CYP2E1,ESR1,GALNT18,HAGH,NADSYN1,NTSR1,PRKAG2,RBFOX3,RETN,RHOJ,RNF220,SLC16A12*	16
	Migration of endothelial cell lines	3.50E−03	*CDH13,CHI3L1,ESR1,MAPK7*	4
	Wolff-Parkinson-White syndrome	4.70E−03	*CACNA1E,PRKAG2,TTC39A*	3
	Sprouting angiogenesis by endothelial cell lines	7.20E−03	*CDH13*	1
	Familial hypertrophic cardiomyopathy-6	7.20E−03	*PRKAG2*	1
	Lethal congenital glycogen storage disease of heart	7.20E−03	*PRKAG2*	1
	Susceptibility to insulin resistance-related hypertension	7.20E−03	*RETN*	1
	Augmented response of HDL cholesterol to hormone replacement	7.20E−03	*ESR1*	1
	Lethal congenital glycogen storage disease of heart	7.20E−03	*PRKAG2*	1
	Susceptibility to insulin resistance-related hypertension	7.20E−03	*RETN*	1

Enriched functional annotations were obtained from an Ingenuity Pathway Analysis (Ingenuity Systems, Redwood City, CA, USA) with an input of genes annotated to DMPs in AF patients in seven groups (*p*-value < 0.01)

*AF*: Atrial fibrillation; *DMP*: Differentially methylated position; *HDL*: High-density lipoprotein

The top canonical pathways when considering all DMPs in the seven AF patient groups are shown in Table 4. Many immune-related complexes and gene-associated pathways were found in four of the seven AF groups (with or without late recurrence, with or without impaired LA function, increased (≥ 45 mm) or decreased (< 45 mm) LA diameter, and increased (≥ 90 ml) or decreased (< 90 ml) LA volume).

**Table 4:** Canonical pathways enriched in differentially methylated datasets (*p*-value < 0.05)

Group	Canonical pathway	−log (*p*-value)	Ratio	Genes
Non-paroxysmal AF (n = 44) vs Paroxysmal AF (n = 71)	Estrogen-mediated S-phase Entry	3.62	0.154	*TFDP1,E2F7,E2F3,ESR1*
Late recurrence ( +) (n = 26) vs Late recurrence (-) (n = 89)	Antigen Presentation Pathway	7.10	0.184	*HLA-DRB1,HLA-A,HLA-C,HLA-B,HLA-DQB1,HLA-F,HLA-DPB1*
	Allograft Rejection Signaling	6.37	0.146	*HLA-DRB1,HLA-A,HLA-C,HLA-B,HLA-DQB1,HLA-F,HLA-DPB1*
	OX40 Signaling Pathway	5.90	0.125	*HLA-DRB1,HLA-A,HLA-C,HLA-B,HLA-DQB1,HLA-F,HLA-DPB1*
	Autoimmune Thyroid Disease Signaling	5.48	0.143	*HLA-DRB1,HLA-A,HLA-C,HLA-B,HLA-DQB1,HLA-F*
	Graft-versus-Host Disease Signaling	5.35	0.136	*HLA-DRB1,HLA-A,HLA-C,HLA-B,HLA-DQB1,HLA-F*
	Cdc42 Signaling	4.37	0.062	*HLA-DRB1,HLA-A,HLA-C,HLA-B,EXOC2,HLA-DQB1,HLA-F,HLA-DPB1*
	Nur77 Signaling in T Lymphocytes	3.76	0.094	*HLA-DRB1,HLA-A,HLA-B,HLA-DQB1,RXRA*
	B Cell Development	3.74	0.138	*HLA-DRB1,HLA-A,HLA-B,HLA-DQB1*
	IL-4 Signaling	3.55	0.066	*HLA-DRB1,HLA-A,HLA-B,IRS2,HLA-DQB1,PIK3R4*
	Th1 Pathway	3.51	0.054	*HLA-DRB1,HLA-A,HLA-B,IRS2,HLA-DQB1,PIK3R4,HLA-DPB1*
	Xenobiotic Metabolism Signaling	3.32	0.036	*AHRR,CES1,ALDH1L2,CHST3,HS6ST3,IRS2,PIK3R4,RXRA,GSTP1,ALDH7A1*
	Th2 Pathway	3.20	0.048	*HLA-DRB1,HLA-A,HLA-B,IRS2,HLA-DQB1,PIK3R4,HLA-DPB1*
	Type I Diabetes Mellitus Signaling	3.17	0.056	*HLA-DRB1,HLA-A,HLA-C,HLA-B,HLA-DQB1,HLA-F*
Impaired left atrial function ( +) (n = 44) vs. Impaired left atrial function (-) (n = 71)	Antigen Presentation Pathway	4.88	0.105	*NLRC5,HLA-C,HLA-B,HLA-F*
	Neuroprotective Role of THOP1 in Alzheimer’s Disease	4.14	0.044	*HLA-C,PRSS22,HLA-B,KLK15,HLA-F*
	Cdc42 Signaling	3.87	0.039	*HLA-C,MYL5,HLA-B,EXOC2,HLA-F*
	Autoimmune Thyroid Disease Signaling	3.26	0.071	*HLA-C,HLA-B,HLA-F*
	Graft-versus-Host Disease Signaling	3.20	0.068	*HLA-C,HLA-B,HLA-F*
	Allograft Rejection Signaling	3.08	0.063	*HLA-C,HLA-B,HLA-F*
	Apelin Cardiomyocyte Signaling Pathway	3.05	0.036	*CAT,MYL5,PLCB3,PIK3R4*
Left atrial diameter (≥ 45 mm) (n = 22) vs Left atrial diameter (< 45 mm) (n = 93)	Netrin Signaling	4.64	0.092	*CACNG6,CACNA1E,PRKG1,CACNA1D,CACNA2D4,CACNA1A*
	nNOS Signaling in Skeletal Muscle Cells	4.59	0.125	*CACNG6,CACNA1E,CACNA1D,CACNA2D4,CACNA1A*
	Antigen Presentation Pathway	3.47	0.105	*HLA-DQB2,HLA-C,HLA-DQB1,HLA-DPB1*
	G Beta Gamma Signaling	3.16	0.050	*CACNG6,CACNA1E,CACNA1D,CAV2,CACNA2D4,CACNA1A*
	Maturity Onset Diabetes of Young Signaling	3.16	0.150	*CACNA1E,CACNA1D,CACNA1A*
	Allograft Rejection Signaling	3.08	0.083	*HLA-DQB2,HLA-C,HLA-DQB1,HLA-DPB1*
	Synaptic Long Term Depression	3.04	0.040	*CACNG6,PRKG1,CACNA1E,CACNA1D,PLA2G4C,CACNA2D4,CACNA1A*
	FcγRIIB Signaling in B Lymphocytes	3.03	0.059	*CACNG6,CACNA1E,CACNA1D,CACNA2D4,CACNA1A*
Left atrial volume (≥ 90 ml) (n = 35) vs. Left atrial volume (< 90 ml) (n = 74)	B Cell Development	4.82	0.172	*HLA-DRB1,HLA-DMB,HLA-DQA1,HLA-DQB1,IL7*
	Antigen Presentation Pathway	4.23	0.132	*HLA-DRB1,HLA-DQB2,HLA-DMB,HLA-DQA1,HLA-DQB1*
	PKCθ Signaling in T Lymphocytes	4.12	0.056	*CACNG6,HLA-DRB1,HLA-DMB,HLA-DQA1,FGFR2,HLA-DQB1,PIK3R4,CACNA2D3,CACNA1A*
	Allograft Rejection Signaling	3.73	0.104	*HLA-DRB1,HLA-DQB2,HLA-DMB,HLA-DQA1,HLA-DQB1*
	Nur77 Signaling in T Lymphocytes	3.53	0.094	*HLA-DRB1,HLA-DMB,HLA-DQA1,HLA-DQB1,RXRA*
	OX40 Signaling Pathway	3.42	0.089	*HLA-DRB1,HLA-DQB2,HLA-DMB,HLA-DQA1,HLA-DQB1*
	IL-4 Signaling	3.28	0.066	*HLA-DRB1,HLA-DMB,HLA-DQA1,FGFR2,HLA-DQB1,PIK3R4*
	Th1 Pathway	3.21	0.054	*HLA-DRB1,HLA-DQB2,HLA-DMB,HLA-DQA1,FGFR2,HLA-DQB1,PIK3R4*
	Netrin Signaling	3.12	0.077	*CACNG6,PRKG1,RYR1,CACNA2D3,CACNA1A*
	nNOS Signaling in Skeletal Muscle Cells	3.01	0.100	*CACNG6,RYR1,CACNA2D3,CACNA1A*
Left atrial flow velocity (≤ 40 cm/sec) (n = 29) vs. Left atrial flow velocity (> 40 cm/sec) (n = 86)	Melanocyte Development and Pigmentation Signaling	3.05	0.049	*ADCY4,PRKAG2,PIK3CD,RPS6KA2,PIK3R4*

Canonical pathway analyses were performed using Ingenuity Pathway Analysis software (Ingenuity Systems, Redwood City, CA, USA) with an input of genes annotated to DMPs in AF patients divided into seven groups (*p*-value < 0.05). Ratio indicates the number of genes annotated to DMPs that map to the pathway to the total number of molecules that map to the canonical pathway. *P*-values were calculated using Fisher’s exact test to generate the probability that the association between genes annotated to DMPs and the canonical pathway was explained by chance alone. *P*-values are expressed as logarithmic values (−log[*p*-value])

**Fig. 2:**
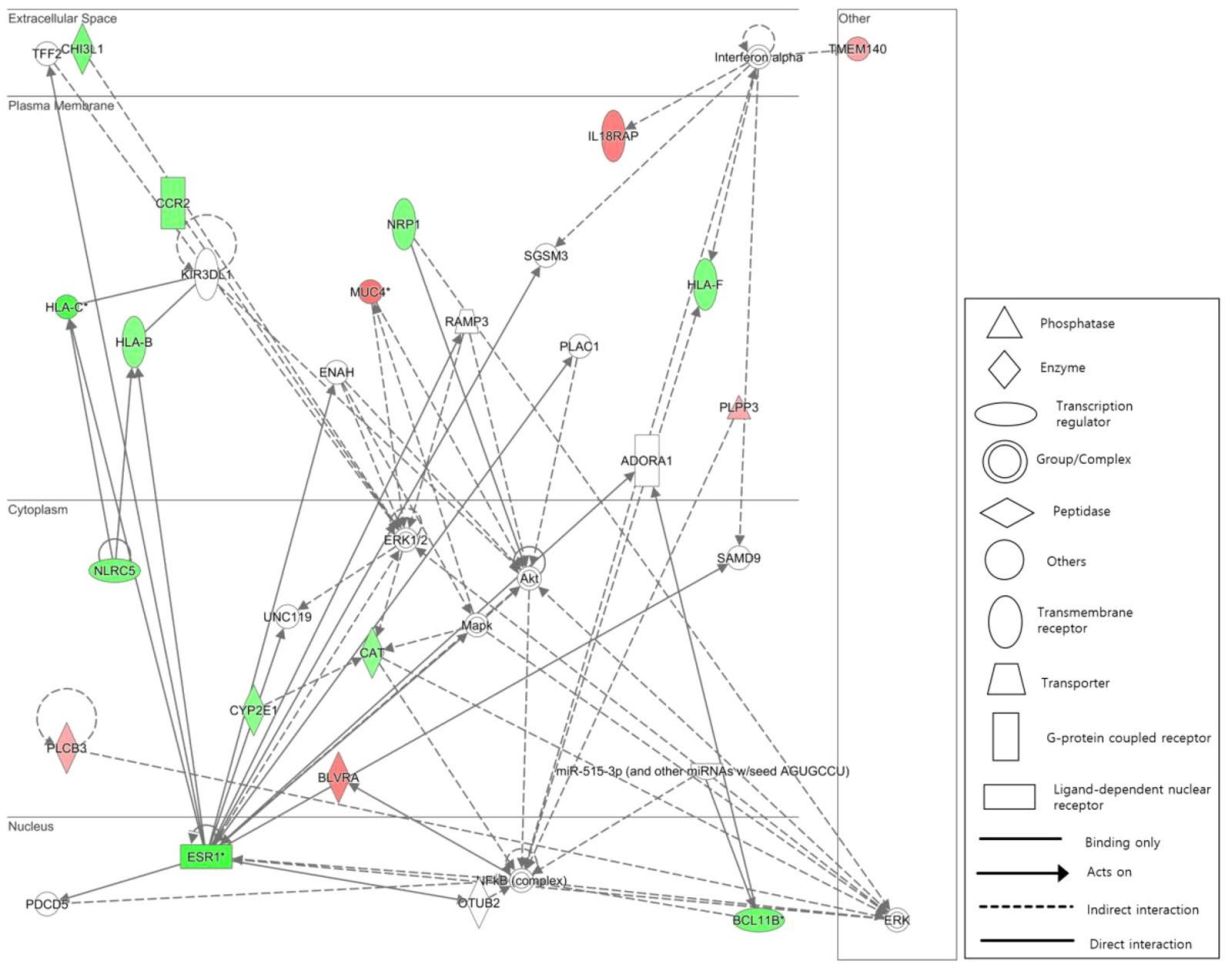
The top network in an Ingenuity Pathway Analysis showing the interaction between genes annotated to DMPs in AF patients with and without impaired LA function. The top functions of this network are endocrine system disorders, gastrointestinal disease, and metabolic disease, with a score of 34. Solid lines between genes represent direct interactions, and dashed lines represent indirect interactions. The shapes of the nodes indicate the functional class of the gene product, and the intensity of the red and green colors indicates the degree of up- and downregulation, respectively. The data were based on blood samples from 115 patients. LA: left atrium

We identified biological networks based on the genes annotated to DMPs in AF patients with and without impaired LA function. This group showed multiple direct or indirect interactions with immune-related complexes and genes, including the NF-κB protein complex, probable regulator of the NF-κB (NLRC5), major histocompatibility complex class I (HLA-B, HLA-F), and interferon alpha, in the top network (Fig. 2). The top functions of this network include endocrine system disorders, gastrointestinal disease, and metabolic disease, with a score of 34.

## Discussion

In this study, we investigated the regulatory effects of DNA methylation in AF by dividing patients into seven groups based on the clinical data. We performed genome‑wide DNA methylation profiling and found altered DNA methylations that were associated with AF. Although identified CpG sites in our study differed from previous studies, we identified 38 DMPs in the seven groups, not only within the CpG islands in the proximal promoter regions of the genes, but also distributed throughout the gene. Seven of the 38 CpG sites were in proximal promoter regions corresponding to five genes.

### DNA methylation and atrial remodeling, and the recurrence of AF

Previous genome-wide methylation studies revealed differentially methylated CpG sites in patient with AF. A study from Offspring Cohort of Framingham Heart study revealed 2 CpG sites and 5 CpG sites associated with prevalent AF and incident AF, respectively [7]. Further methylation analysis of left atrial tissue from permanent AF showed 417 differentially methylated CpG sites, which participated in the activation of inflammation, sodium and potassium ion transport, fibrosis and the reduction of lipid metabolism [19].

DNA methylation is an important epigenetic mechanism that contribute to the development of AF. Heart failure could enhance hypermethylation of PITX2c promoter region and increase DNA methyltransferase, which lead to decreased PITX2c expression of left atrium and increase of cardiac arrhythmias [20, 21]. DNA methylation has also been suggested to play a role in cardiac inflammation and fibroblast activation [22]. Hypermethylation of DNA suppresses antiproliferative and anti-myofibroblast differentiation genes in human heart, which accelerates cardiac fibrosis [23, 24]. Interestingly, cg20223677, which is located within the promoter of DEFB104B, was found to be associated with childhood asthma [25]. Persistent asthma has been reported to be associated with increased risk of AF, which could be driven by increase of AF triggers or substrates [26]. We were able to replicate the differential methylation at cg20223677 through pyrosequencing regarding LA function, implying that alteration of DNA methylation in DEFB104B may lead to accelerated atrial structural remodeling. Also, hypomethylation of cg20223677 was observed in AF patients with late recurrence, impaired LA function, and impaired flow velocity of the LAA. Late recurrence after RFCA is significantly affected by atrial myopathy, which is expressed as increased LA diameter and decreased LAA flow velocity [13]. Therefore, our findings suggest that hypomethylation of cg20223677 is associated with progressed atrial myopathy, which is significantly linked to late recurrence after catheter ablation.

### Inflammation and immune response and AF

Although the identified genes in our study were not established to be associated with AF, we found a significant proportion of genes related to the regulation of immune response. The TANC1 gene regulates dendritic spine and excitatory synapses, which is highly expressed in human hearts [27]. Genome-wide association study revealed significant association of TANC1 with sudden cardiac death, and TANC1 hypermethylation was reported in anti-tuberculosis drug-induced liver injury [27, 28]. C3 is a protein coding gene crucial for the activation of complement system. TMEM9B regulates the inflammatory signaling pathways, and increased expression of TMEM9B was found in coronary tissue of sudden coronary death [29]. The DEFB104B gene encodes β-defensin 104B, which has antimicrobial activity and various functions in innate and adaptive immunity [30]. β-defensins are produced by epithelial cells in many organs and have been shown to have impaired function in pulmonary inflammation [31].

Immune response has been suggested to play pathologic roles in AF development [32]. T cells are known to contribute to AF, mainly by regulating the innate immune response, and B cells might have effects by secreting autoantibodies. Tumor Necrosis Factor-α—a proinflammatory cytokine that is increased in failing heart—enhances promoter methylation of SERCA2a and leads to cardiac systolic and diastolic dysfunction relevant to AF [24]. Furthermore, comorbid immune-related disease such as heart failure or coronary artery disease could predispose to the pathogenesis of AF. In turn, AF can exacerbate the immune response, leading to a vicious cycle. We have identified novel DNA-methylated genes and immune-related pathways that correlate with AF. Our findings strengthen the evidence for an association between AF and the immune and inflammatory response.

### Clinical implication

Antiarrhythmic drug and catheter ablation for AF have evolved through decades, but the efficacy remains suboptimal. In addition, pulmonary vein isolation is the only generalized ablation strategy, and there is no other proven treatment strategy for AF that persists after pulmonary vein isolation. Epigenomic regulation provides the mechanism for the pathogenesis of AF that precedes to atrial remodeling. Several identified CpG sites in our study was associated with impaired left atrial function, which increase the risk of AF recurrence after catheter ablation. Furthermore, enriched functional annotation of the differentially methylated sites implies that dysregulation in angiogenesis and endothelial function may be linked to the AF pathogenesis. Endothelial dysfunction is commonly observed in patients with AF and is associated with poor clinical outcome [33]. In this regard, understanding DNA methylation not only elaborates the mechanism of epigenetic regulation of gene expression in the development of AF, but helps to find biomarker for predicting prognosis and response to therapy [34]. Epigenetics may also offer potential therapeutic targets for the individualized treatment of AF. Although epidrugs are currently limited for the treatment of cancer, they could potentially be considered for the treatment of AF [35]. DNA methyltransferase inhibitors lead to the reversal of DNA hypermethylation and restoration of sinus rhythm [36]. In an animal model, treatment with decitabine has been reported to improve atrial tachycardia and left ventricular fibrosis [37]. Similarly, hypomethylating agents to reverse cardiac fibroblast activation could be the promising therapy for AF and advanced atrial myopathy [22]. Further epigenetic therapy will be a promising approach in the management of AF, which may provide a personalized and predictive medicine.

### Limitations

This study has several limitations. First, since an integrated genome-wide DNA methylation and gene expression analysis had not been performed, we were unable to assess the effects of methylation on gene expression regulation. Gene expression data were not generated in our study, and further validation using public gene expression data was not available due to limited sample size in Gene Expression Omnibus database. However, bisulfite pyrosequencing was used for validation, which supports the quantitative analysis of DNA methylation [38, 39]. Second, blood sampling was performed at a fixed time period (before catheter ablation), and follow-up sampling was not conducted after catheter ablation. Currently, there is no study that focused on the epigenetic change after restoration of sinus rhythm. Exploring the dynamic change of epigenetics after catheter ablation and its correlation with AF recurrence may expand the clinical utility of epigenome as biomarkers for treatment response. Similarly, the clinical characteristics of AF were obtained at baseline, and the longitudinal change of AF severity was not assessed. In addition, outcome such as late recurrence is not the absolute predictor of AF progression. The changing paradigm from recurrence defined as a single episode of AF to quantitative measurement of AF burden should be acknowledged in further studies [40]. Lastly, the generalizability of our results is limited in Korean AF patients treated with RFCA. A relatively small sample size is another limitation of our study. Genetic factors and epigenetics differ according to ethnicities, which influence the AF occurrence and consequences. Our analysis represents a unique epigenetics of Korean descents, which was also focused on the spectrum of AF eligible for rhythm control. Further analysis on multiple ethnicities may indicate the common genomic region.

## Conclusion

Epigenetic profiling revealed molecular differences among seven groups of AF patients that could contribute to differential phenomena among patients with AF. Fundamental characteristics of AF, such as atrial myopathy and the response to RFCA, could be affected by epigenetic modulation. The identified methylation-susceptible loci might be associated with the initiation and progression of AF and might therefore be used in targeted therapies, including the use of epigenetic-based therapeutic agents.

## Data Availability

The data underlying this article are available in the article. The raw data underlying this article cannot be shared publicly due to privacy reasons and legal regulations of Republic of Korea.

## Abbreviations

AF: Atrial fibrillation
CpG: Cytosine-phosphate-guanine
DMP: Differentially methylated position
LA: Left atrium
LAA: Left atrial appendage
RFCA: Radiofrequency catheter ablation
